# Supplementary material for: Social support coping strategies among sub-Saharan African refugees: A systematic review and meta-synthesis
Source: Glob Ment Health (Camb). 2026 Feb 12;13:e43. doi: 10.1017/gmh.2026.10150 (PMC12973250; doi:10.1017/gmh.2026.10150)
Supplement: Gebeyehu et al. supplementary material 1 — Gebeyehu et al. supplementary material [file S2054425126101502sup001.docx]

| **Section and Topic** | **Item #** | **Checklist item** | **Location where the item is reported (Page)** |
| --- | --- | --- | --- |
| **TITLE** | Page # |  |  |
| Title | 1 | Identify the report as a systematic review. | Page 1 |
| **ABSTRACT** |  |  |  |
| Abstract | 2 | See the PRISMA 2020 for Abstracts checklist. | Abstract document |
| **INTRODUCTION** |  |  |  |
| Rationale | 3 | Describe the rationale for the review in the context of existing knowledge. | Introduction Page 1-2 |
| Objectives | 4 | Provide an explicit statement of the objective(s) or question(s) the review addresses. | Introduction Page 2 |
| **METHODS** |  |  |  |
| Information sources | 5 | Specify all databases, registers, websites, organisations, reference lists, and other sources searched or consulted to identify studies. Specify the date when each source was last searched or consulted. | Methods Page 2 |
| Search strategy | 6 | Present the full search strategies for all databases, registers, and websites, including any filters and limits used. | Methods Page 3 |
| Eligibility criteria | 7 | Specify the inclusion and exclusion criteria for the review and how studies were grouped for the syntheses. | Methods Page 4 |
| Selection process | 8 | Specify the methods used to decide whether a study met the inclusion criteria of the review, including how many reviewers screened each record and each report retrieved, whether they worked independently, and, if applicable, details of automation tools used in the process. | Methods Page 4 |
| Data collection process | 9 | Specify the methods used to collect data from reports, including how many reviewers collected data from each report, whether they worked independently, any processes for obtaining or confirming data from study investigators, and, if applicable, details of automation tools used in the process. | Methods Page 4 |
| Data items | 10a | List and define all outcomes for which data were sought. Specify whether all results compatible with each outcome domain in each study were sought (e.g., for all measures, time points, and analyses), and if not, the methods used to determine which results to collect. | Page 4 |
|  | 10b | List and define all other variables for which data were sought (e.g., participant and intervention characteristics, funding sources). Describe any assumptions made about any missing or unclear information. | N/A |
| Study risk of bias assessment | 11 | Specify the methods used to assess risk of bias in the included studies, including details of the tool(s) used, how many reviewers assessed each study, and whether they worked independently, and if applicable, details of automation tools used in the process. | Methods Page 5 |
| Effect measures | 12 | Specify the effect measure(s) (e.g., risk ratio, mean difference) used in the synthesis or presentation of results for each outcome. | N/A |
| Synthesis methods | 13a | Describe the processes used to decide which studies were eligible for each synthesis (e.g., tabulating the study intervention characteristics and comparing against the planned groups for each synthesis (item #5)). | Page 5 |
|  | 13b | Describe any methods required to prepare the data for presentation or synthesis, such as handling of missing summary statistics or data conversions. | Page 5 |
|  | 13c | Describe any methods used to tabulate or visually display the results of individual studies and syntheses. | Page 5 |
|  | 13d | Describe any methods used to synthesise results and provide a rationale for the choice(s). If a meta-analysis was performed, describe the model(s), method(s) used to identify the presence and extent of statistical heterogeneity, and the software package(s) used. | Page 6-7 |
|  | 13e | Describe any methods used to explore possible causes of heterogeneity among study results (e.g., subgroup analysis, meta-regression). | N/A |
|  | 13f | Describe any sensitivity analyses conducted to assess the robustness of the synthesised results. | N/A |
| Reporting bias assessment | 14 | Describe any methods used to assess the risk of bias due to missing results in a synthesis (arising from reporting biases). | N/A |
| Certainty assessment | 15 | Describe any methods used to assess certainty (or confidence) in the body of evidence for an outcome. | N/A |
| **RESULTS** |  |  |  |
| Study selection | 16a | Describe the results of the search and selection process, from the number of records identified in the search to the number of studies included in the review, ideally using a flow diagram. | Results Page 7 |
|  | 16b | Cite studies that might appear to meet the inclusion criteria, but which were excluded, and explain why they were excluded. | Page 7 and  Figure 1 |
| Study characteristics | 17 | Cite each included study and present its characteristics. | Results Page 7- 8 &  Table 2 (Tables document) |
| Risk of bias in studies | 18 | Present assessments of risk of bias for each included study. | Results Page 8 |
| Results of individual studies | 19 | For all outcomes, present, for each study: (a) summary statistics for each group (where appropriate) and (b) an effect estimate and its precision (e.g., confidence/credible interval), ideally using structured tables or plots. | Table 1 (Tables document) |
| Results of syntheses | 20a | For each synthesis, briefly summarise the characteristics and risk of bias among contributing studies. | Results Page 8 &  Table 3 (Tables document) |
|  | 20b | Present the results of all statistical syntheses conducted. If a meta-analysis was performed, present the summary estimate and its precision (e.g., confidence or credible interval) for each, along with measures of statistical heterogeneity. If comparing groups, describe the direction of the effect. | Results Page 8-19  Figure 2 |
|  | 20c | Present the results of all investigations of possible causes of heterogeneity among study results. | N/A |
|  | 20d | Present the results of all sensitivity analyses conducted to assess the robustness of the synthesised results. | N/A |
| Reporting biases | 21 | Present assessments of risk of bias due to missing results (arising from reporting biases) for each synthesis assessed. | N/A |
| Certainty of evidence | 22 | Present assessments of certainty (or confidence) in the body of evidence for each outcome assessed. | N/A |
| **DISCUSSION** |  |  |  |
| Discussion | 23a | Provide a general interpretation of the results in the context of other evidence. | Discussion Page 19-23 |
|  | 23b | Discuss any limitations of the evidence included in the review. | Page 24-25 |
|  | 23c | Discuss any limitations of the review processes used. | Page 24-25 |
|  | 23d | Discuss implications of the results for practice, policy, and future research. | Discussion Page 23-24 |
| **OTHER INFORMATION** |  |  |  |
| Registration and protocol | 24a | Provide registration information for the review, including the register name and registration number, or state that the review was not registered. | Methods Page 3 |
|  | 24b | Indicate where the review protocol can be accessed, or state that a protocol was not prepared. | Methods Page 3 |
|  | 24c | Describe and explain any amendments to information provided at registration or in the protocol. | N/A |
| Support | 25 | Describe sources of financial or non-financial support for the review, and the role of the funders or sponsors in the review. | Declarations Page 26 |
| Competing interests | 26 | Declare any competing interests of review authors. | Declarations Page 26 |
| Availability of data, code, and other materials | 27 | Report which of the following are publicly available and where they can be found: template data collection forms; data extracted from included studies; data used for all analyses; analytic code; any other materials used in the review. | Declarations Page 26 |

*From:*  Page MJ, McKenzie JE, Bossuyt PM, Boutron I, Hoffmann TC, Mulrow CD, et al. The PRISMA 2020 statement: an updated guideline for reporting systematic reviews. BMJ 2021;372:n71. doi: 10.1136/bmj.n71. This work is licensed under CC BY 4.0. To view a copy of this license, visit <https://creativecommons.org/licenses/by/4.0/>
