# Supplementary material for: Social support coping strategies among sub-Saharan African refugees: A systematic review and meta-synthesis
Source: Glob Ment Health (Camb). 2026 Feb 12;13:e43. doi: 10.1017/gmh.2026.10150 (PMC12973250; doi:10.1017/gmh.2026.10150)
Supplement: Gebeyehu et al. supplementary material 2 — Gebeyehu et al. supplementary material [file S2054425126101502sup002.docx]

Supplementary table S1: Data searching subject headings/MeSH terms and keywords

|  | **Subject Headings/ Mesh terms** | **Key words** | **Remarks** |
| --- | --- | --- | --- |
| #1 | “Adaptation, psychological”[MeSH:NoExp] | “Coping strategies” OR “coping behaviour” OR “coping skills” OR "coping mechanisms" OR coping OR cope* OR adapt* OR “Psychological Coping” OR social* " OR “support, social” OR “mental health” |  |
| #2 | "Conflict, psychological"[MeSH] “Armed conflicts”[MeSH:NoExp] | "Armed conflict" OR Conflict OR war OR trauma* OR torture OR Migration OR displace* |  |
| #3 | Sub-Saharan African People[MeSH] | "Sub-Saharan African" OR Angola OR Benin OR Botswana OR "Burkina Faso" OR Burundi OR Cameroon OR "Central African" OR Chad OR Congo OR "Cote D’Ivoire" OR Djibouti OR Equatorial guinea OR Eritrea OR Eswatini OR Ethiopia OR Gabon OR Gambia OR Ghana OR Guinea OR Guinea-Bissau OR Kenya OR Liberia OR Malawi OR Mali OR Mauritania OR Mozambique OR Namibia OR Niger OR Nigeria OR Rwanda OR Senegal OR "Sierra Leone" OR Somalia OR "south Africa" OR south Sudan OR Sudan OR Tanzania OR Togo OR Uganda OR Zambia OR Zimbabwe OR "Eastern Africa" OR "Western Africa" OR African |  |
| #4 | Refugees[MeSH] | Refugee* OR Migrant* OR displace* OR resettlement |  |
| #5 | "Developed countries"[MeSH] | "High-Income Countries" OR "Western Countries" OR Resettlement OR adaptation OR "United States" OR Canada OR United Kingdom OR Germany OR France OR Australia OR Sweden OR Norway OR Switzerland OR Netherlands OR Denmark OR Belgium OR Austria OR Finland OR Luxembourg OR Iceland OR Ireland OR Europe |  |

Note: The subject headings presented here are derived from all the databases we used for the search.

**Sub-Saharan African countries**: are the countries located south of the Sahara desert, such as Angola, Benin, Botswana, Burkina Faso, Burundi, Cameroon, Central Africa, Chad, Congo, Cote d'Ivoire, Djibouti, Equatorial Guinea, Eritrea, Eswatini, Ethiopia, Gabon, Gambia, Ghana, Guinea, Guinea-Bissau, Kenya, Liberia, Malawi, Mali, Mauritania, Mozambique, Namibia, Niger, Nigeria, Rwanda, Senegal, Sierra Leone, Somalia, South Africa, South Sudan, Sudan, Tanzania, Togo, Uganda, Zambia, and Zimbabwe.

**High-income countries** are defined as developed countries with a per capita income, according to the World Bank, where African refugees are often resettled. These countries are the United States, Canada, the United Kingdom, Germany, France, Australia, Sweden, Norway, Switzerland, the Netherlands, Denmark, Belgium, and Finland.
